# Identification of the BolA Protein Reveals a Novel Virulence Factor in K. pneumoniae That Contributes to Survival in Host

**DOI:** 10.1128/spectrum.00378-22

**Published:** 2022-09-19

**Authors:** Feiyang Zhang, Xiangjin Yan, Jiawei Bai, Li Xiang, Manlin Ding, Qin Li, Biying Zhang, Qinghua Liang, Yingshun Zhou

**Affiliations:** a Department of Pathogen Biology, School of Basic Medicine, Public Center of Experimental Technology of Pathogen Biology Technology Platform, Southwest Medical University, Luzhou, China; b Department of Clinical Laboratory, Zigong First People’s Hospital, Zigong, China; Peking University People's Hospital

**Keywords:** *Klebsiella pneumoniae*, BolA, siderophore, virulence, colonization

## Abstract

BolA has been characterized as an important transcriptional regulator, which is induced in the stationary phase of growth and is often associated with bacterial virulence. This study was initiated to elucidate the role of the BolA in the virulence of K. pneumoniae. Using a mouse infection model, we revealed *bolA* mutant strain yielded significantly decreased bacterial loads in the liver, spleen, lung, and kidney, and failed to form liver abscesses. Gene deletion demonstrated that the *bolA* was required for siderophore production, biofilm formation, and adhesion to human colon cancer epithelial cells HCT116. Quantitative reverse transcriptase PCR (RT-qPCR) indicated that BolA could impact the expression of *pulK*, *pulF*, *pulE*, *clpV*, *vgrG*, *entE*, *relA*, and *spoT* genes on a genome-wide scale, which are related to type II secretion system (T2SS), type VI secretion system (T6SS), guanosine tetraphosphate (ppGpp), and siderophore synthesis and contribute to fitness in the host. Furthermore, the metabolome analysis showed that the deletion of the *bolA* gene led to decreased pools of five metabolites: biotin, spermine, cadaverine, guanosine, and flavin adenine dinucleotide, all of which are involved in pathways related to virulence and stress resistance. Taken together, we provided evidence that BolA was a significant virulence factor in the ability of K. pneumoniae to survive, and this was an important step in progress to an understanding of the pathways underlying bacterial virulence.

**IMPORTANCE** BolA has been characterized as an important transcriptional regulator, which is induced in the stationary phase of growth and affects different pathways directly associated with bacterial virulence. Here, we unraveled the role of BolA in several phenotypes associated with the process of cell morphology, siderophore production, biofilm formation, cell adhesion, tissue colonization, and liver abscess. We also uncovered the importance of BolA for the success of K. pneumoniae infection and provided new clues to the pathogenesis strategies of this organism. This work constitutes a relevant step toward an understanding of the role of BolA protein as a master regulator and virulence factor. Therefore, this study is of great importance for understanding the pathways underlying K. pneumoniae virulence and may contribute to public health care applications.

## INTRODUCTION

Klebsiella pneumoniae is an important opportunistic pathogen that is often responsible for nosocomial infections, including urinary tract infection, pneumonia, and bloodstream infection (1–5). However, the underlying factors in K. pneumoniae to efficiently infect and survive inside the host are still unclear (6). To combat these daunting infections, it is necessary to determine the virulence factor of K. pneumoniae.

Stress response protein BolA affects different pathways directly related to bacterial virulence, which was first discovered in Escherichia coli, and its homologs form a broadly conserved family of proteins in prokaryotes and eukaryotes (7–9). In Gram-negative bacteria, the expression of BolA is induced due to the depletion of nutrients or in response to several stresses which leads to a short spherical morphology and allows bacteria to quickly adapt to hostile conditions (10–12). BolA has pleiotropic effects and controls a variety of phenotypes like outer membrane permeability, Fe-S cluster biogenesis, and iron sensing and regulation (13–15). Characterized as a transcription factor, BolA also participates in the expression of diguanylate cyclases (DGCs) and phosphodiesterases (PDEs), affects the synthesis and degradation of the secondary signal metabolite c-di-GMP, induces the expression of tricarboxylic acid (TCA) cycle genes, fimbria-like adhesins-related genes, represses the expression of flagellum-associated genes with consequences for bacterial motility and biofilm formation (16, 17). All these adaptation processes confer specific advantages for bacterial survival and are often associated with virulence.

To date, the role of BolA protein in the induction of important stress-resistant physiological changes and its recent implication in virulence have been well characterized in E. coli, Salmonella enterica serovar Typhimurium, and other species of bacteria (7, 12, 18). However, the role of BolA protein in the virulence of K. pneumoniae has not yet been characterized. These studies hinted that BolA might play an important role in bacterial virulence and prompted us to investigate the role of BolA in the virulence of K. pneumoniae to understand and overcome K. pneumoniae strategies for successful infection.

In the present work, we identified a BolA homolog in K. pneumoniae NTUH-K2044 and unraveled its role in several phenotypes associated with the virulence of K. pneumoniae. We tested the growth rate of K. pneumoniae NTUH-K2044 and the *bolA* mutant strains in M9 and LB medium, the ability of iron uptake, the adhesion to HCT116 cells, and their resistance to bile, osmotic and oxidative stress, as well as the ability to kill Galleria mellonella larvae. The colonization ability of different strains in the liver, lung, spleen, and kidney tissues of mice and the role of BolA in liver abscess formation were also tested by the mice septicemia infection model.

## RESULTS

### Identification of E. coli and *S.* Typhimurium BolA homologs in K. pneumoniae.

**FIG 1:**
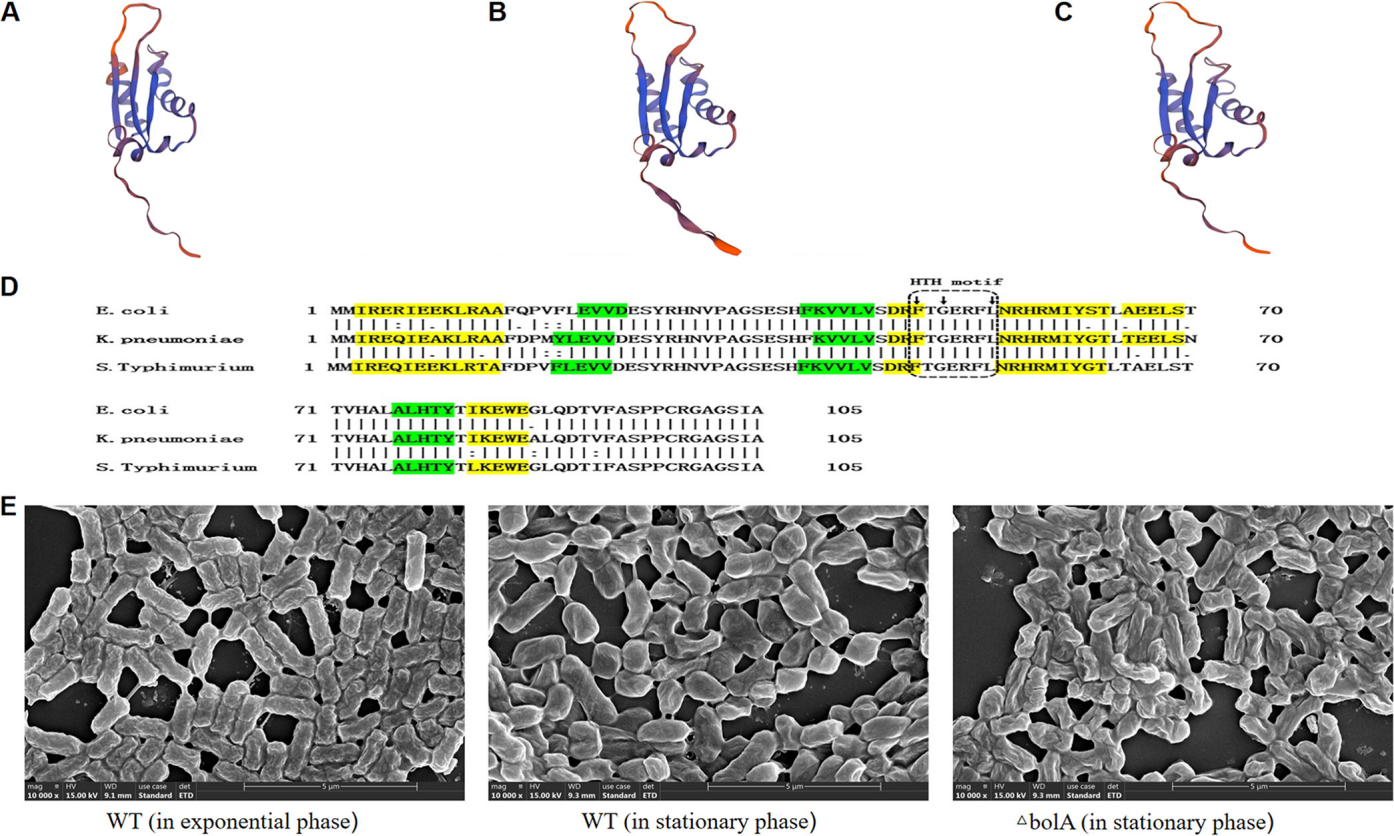
The 3D structure computer model and multiple sequence alignments of BolA protein, and the SEM images of the WT and the *bolA* mutant strains. (A) A computer model of the 3D structure predicted from K. pneumoniae NTUH-K2044 BolA protein using SWISS-MODEL is presented. (B and C) The computer models of the 3D structure predicted from E. coli K-12 W3110 and *S.* Typhimurium SL1344 BolA protein using SWISS-MODEL are presented, respectively. (D) Multiple sequence alignment of BolA from E. coli K-12 W3110, *S.* Typhimurium SL1344, and K. pneumoniae NTUH-K2044 were obtained with the DNAMAN software. In green are the β-sheets and in yellow are the α-helixes. The small arrows at the top of the sequence represent the amino acid of the characteristic helix to helix (HTH) FXGXXXL sequence. (E) The SEM images of the bacterial cell morphology are displayed (including the WT and *bolA* mutant strains).

The E. coli K-12 W3110 amino acid sequence (GenBank accession no. APC50728.1), *S.* Typhimurium SL1344 amino acid sequence (GenBank accession no. FQ312003.1), and K. pneumoniae NTUH-K2044 amino acid sequence (laboratory sequencing results) were used to search for the BolA homologs in K. pneumoniae. The predicted three-dimensional (3D) structures of the BolA proteins were nearly identical for the three species (Fig. 1A to C). Multiple sequence alignments of BolA from E. coli, *S.* Typhimurium, and K. pneumoniae were obtained with the DNAMAN software, and the BolA sequence of K. pneumoniae and E. coli had 91.4% similarity, and that of *S.* Typhimurium SL1344 was 91.4% (Fig. 1D). It was found that K. pneumoniae BolA protein had an α1β1β2α2α3β3α4 fold with four α-helices and three β-sheets. The helix-turn-helix (HTH) signature FXGXXXL sequence, characteristic of BolA proteins and typically implicated in protein-DNA interaction, was also present.

### BolA was required for maintaining cell morphology and did not affect the growth rate of K. pneumoniae.

We used a hypervirulent K. pneumoniae strain (NTUH-K2044, the wild-type [WT] strain) to construct the *bolA* mutant strain (Δ*bolA*) and the complementation strain (Δ*bolA+bolA*). The scanning electron microscopy (SEM) of these cells revealed the effects of *bolA* on cell morphology (Fig. 1E). During the exponential phase, it was found that WT cells exhibited the classical rod-shaped morphology. However, in the stationary phase, WT cells showed a shorter and wider morphology, while the Δ*bolA* cells were generally longer and thinner than the WT cells and were similar to the shape of WT cells during the exponential phase. Because BolA was identified as a stress-responsive protein, we then explored the growth rate of WT and Δ*bolA* strains in nutrient-rich LB and nutrient-restricted M9 medium. The results showed that the deletion of the *bolA* gene did not affect the growth rate of K. pneumoniae in the LB medium, and the final concentrations of the WT and Δ*bolA* strains were almost identical. In the M9 medium, the growth rate of the Δ*bolA* strain was reduced compared with the WT strain, and the final concentration of the Δ*bolA* strain was also decreased. However, there was no significant difference in growth rate between these two strains (*P* > 0.05). The growth curves of the WT and the Δ*bolA* strains are shown in Fig. S1 in Supplemental File 1.

### BolA positively regulated siderophores production and biofilm formation.

**FIG 2:**
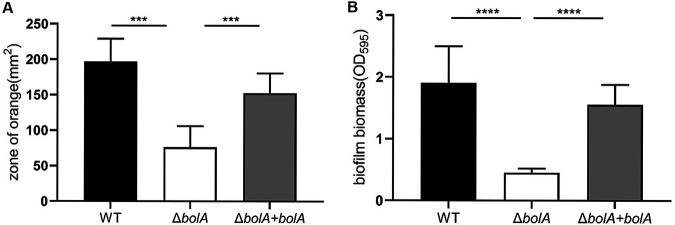
The role of BolA in the production of siderophores and biofilm formation. (A) CAS plates were used to measure the production of siderophores. The area of the orange halo in the CAS agar plate was determined after 48 h of cultivation at 28°C. The larger area of the orange halo indicates that the strain has produced more siderophores and increased the capacity of iron uptake. (B) The 96-well plate assay results showed K. pneumoniae *bolA* mutant strain was defective in biofilm formation. After 48 h of incubation, the biofilm biomass was measured using a 0.1% of crystal violet assay, and the optical density was determined at 595 nm. ***, *P* < 0.001; ****, *P* < 0.0001 by *t* test.

Chrome azurol S (CAS) agar assays were used to detect the siderophores of K. pneumoniae. It was observed that the WT strain exhibited a larger orange halo than the Δ*bolA* strain. In other words, the *bolA* deletion reduced the area of the orange halo by about 2.6 times, which was statistically significant (*P* < 0.001). The ability to produce siderophores of the Δ*bolA+bolA* strain has nearly recovered to the level of the WT strain (Fig. 2A). This result suggested that the deletion of the *bolA* gene reduced the production of siderophores. We then investigated whether K. pneumoniae BolA affected biofilm formation. Using a 96-well plate model, we found that the biofilm biomass of the Δ*bolA* strain was significantly lower than the WT strain. The Δ*bolA+bolA* strain nearly restored its biofilm biomass, which was statistically significant (*P* < 0.001) (Fig. 2B), suggesting that BolA was required for K. pneumoniae biofilm formation.

### BolA effectively regulated the expression of T2SS, T6SS, siderophores, and iron transport-related genes.

**FIG 3:**
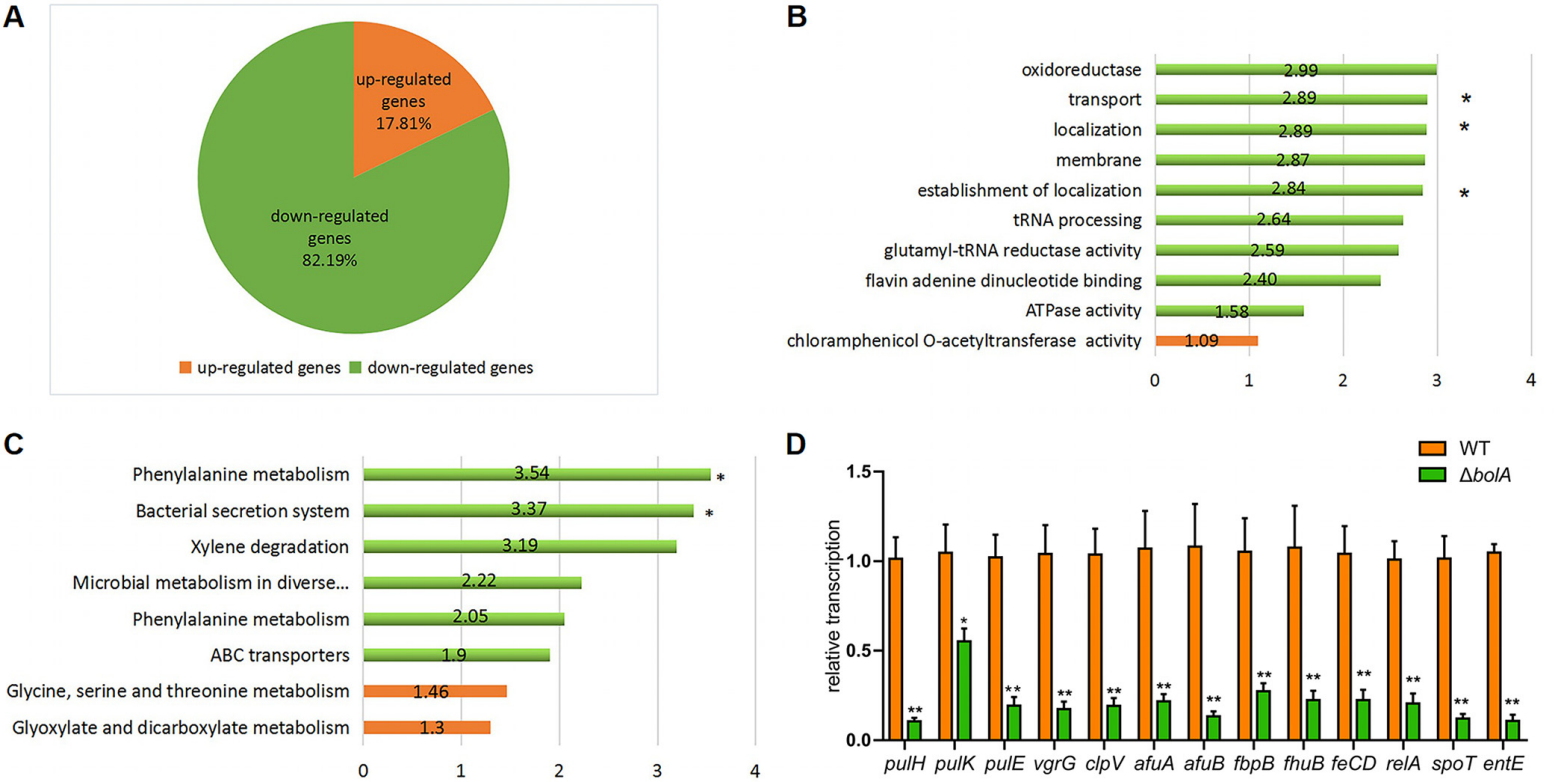
Transcriptome analysis between the WT and Δ*bolA* strains. (A) Pie chart of 146 genes significantly regulated by *bolA* gene. The number of genes significantly downregulated or upregulated is indicated in green and orange, respectively. (B) Graphical representation of the gene GO enrichment analysis was in “biological process,’’ “cellular component,” and “molecular function” categories for genes downregulated or upregulated in response to the *bolA* gene. *, *P* value associated with the enrichment test was lower than 1 × 10^−4^. No asterisk indicates that the *P* value associated with the enrichment test was between 1 × 10^−2^ and 1 × 10^−4^. (C) Functional classification of KEGG pathway. The KEGG enrichment pathways were summarized in eight main pathways. *, *P* value associated with the enrichment test was lower than 0.01. No asterisk indicates that the *P* value associated with the enrichment test was between 0.01 and 0.05. In all, the bar length corresponds to the average log_2_ change (in the absolute value of the Δ*bolA/*WT ratio) of the genes significantly upregulated or downregulated associated with each KEGG or GO enrichment pathway, and the red graphic shows upregulated pathways and the green the downregulated pathways. (D) Results of the expression of genes related to T2SS, T6SS, siderophores, and ppGpp synthesis between WT and Δ*bolA* strains. The expression levels of these genes were detected by RT-qPCR, and the experiments were carried out in triplicate with three independent RNA samples. Student's *t* test with GraphPad Prism software was used to analyze the continuous data obtained by RT-qPCR. *, *P* < 0.05; **, *P* < 0.01.

RNA-sequencing was performed to compare the transcriptomic profiles of the WT and Δ*bolA* strains, and a total of 146 differentially expressed genes were identified between these two strains in the stationary phase. Among these differentially expressed genes, 26 genes were upregulated, and 120 genes were downregulated. That is, the overwhelming majority of differentially expressed genes were downregulated (Fig. 3A). Software GOseq based on Wallonia’s noncentral hypergeometric distribution was performed on the groups of genes significantly upregulated or downregulated, among which, the downregulated differentially expressed genes were mainly enriched in T2SS and T6SS, and these two secretion systems were important virulence factors for bacteria. Gene ontology (GO) enrichment analysis showed differentially expressed genes involved in the membrane, localization, transport, oxidoreductase, and tRNA processing (Fig. 3B). Kyoto Encyclopedia of Genes and Genomes (KEGG) enrichment analysis indicated differentially expressed genes involved in metabolism, transporters, and secretion system (Fig. 3C). As reported previously, stress response protein BolA was confirmed to be induced during the transition into the stationary-phase (7, 19). It was interesting to observe that the genes in the Δ*bolA* strain were downregulated during the stationary phase (Table 1), including several genes (*pulK*, *pulF*, *pulE*, *pulH*, *clpV*, *tssH*, and *vgrG*) associated with the secretion system. More surprisingly, the expression of the gene associated with enterobactin synthesis (*entE*) and several genes associated with iron transport, including *KP1_3194*, *fbpB* (*KP1_3174*), *fecD*, *fhuB* (*KP1_1440*), *afuA*, and *afuB* were downregulated during the stationary-phase. BolA also promoted the expression of several biofilm-related genes (*mhpB*, *ydeY*, and *iolD*), oxidoreduction-related genes (*ulaA*, *KP1_4425*, *KP1_1420* and *KP1_2565*) and K^+^ transport genes (*kdpA* and *kdpB*).

**TABLE 1:** Differentially expressed genes involved in different pathways^*a*^

Gene	log_2_ fold change (Δ*bolA*/WT)	*P* value
Secretion system genes		
*pulK*	−2.9395	0.00683
*pulF*	−2.6985	0.00020409
*pulH*	−4.4938	0.0026801
*pulE*	−5.4198	1.76E−05
*clpV*	−1.6083	0.001354
*vgrG*	−3.0464	0.0017717
Biofilm genes		
*mhpB*	−4.4938	0.0026801
*ydeY*	−2.3545	0.00017415
*iolD*	−1.0834	0.00010384
Oxidoreductase genes		
*ulaA*	−4.3418	0.0046157
*KP1*_*4425*	−3.4869	0.00011244
*KP1*_*1420*	−2.4485	3.50E−06
*KP1*_*2565*	−1.824	0.0021176
K transport genes		
*kdpA*	−1.824	0.0021176
*kdpB*	−4.1932	8.04E−08
Iron transport genes		
*fhuB*	−2.3265	0.0027437
*fecD*	−1.0755	0.0022485
*fbpB*	−1.3265	2.57E−05
*afuA*	−2.3265	0.0027437
*afuB*	−3.9395	1.70E−06
*KP1_3194*	−1.1371	3.85E−05
Enterobactin synthesis gene		
*entE*	−1.7892	5.32E–06

a The value of log_2_ fold change was negative, which means these genes were down-regulated after the *bolA* was deleted.

To validate the results obtained in RNA-sequencing, the expression levels of several genes were further analyzed by RT-qPCR (Fig. 3D). Thirteen different genes were selected for analysis, including the differentially expressed genes mentioned above and ppGpp synthetic-related genes. The regulation patterns of these genes were consistent with the RNA-sequencing results and were downregulated after *bolA* deletion.

### BolA regulated the metabolites related to stress resistance and virulence.

**FIG 4:**
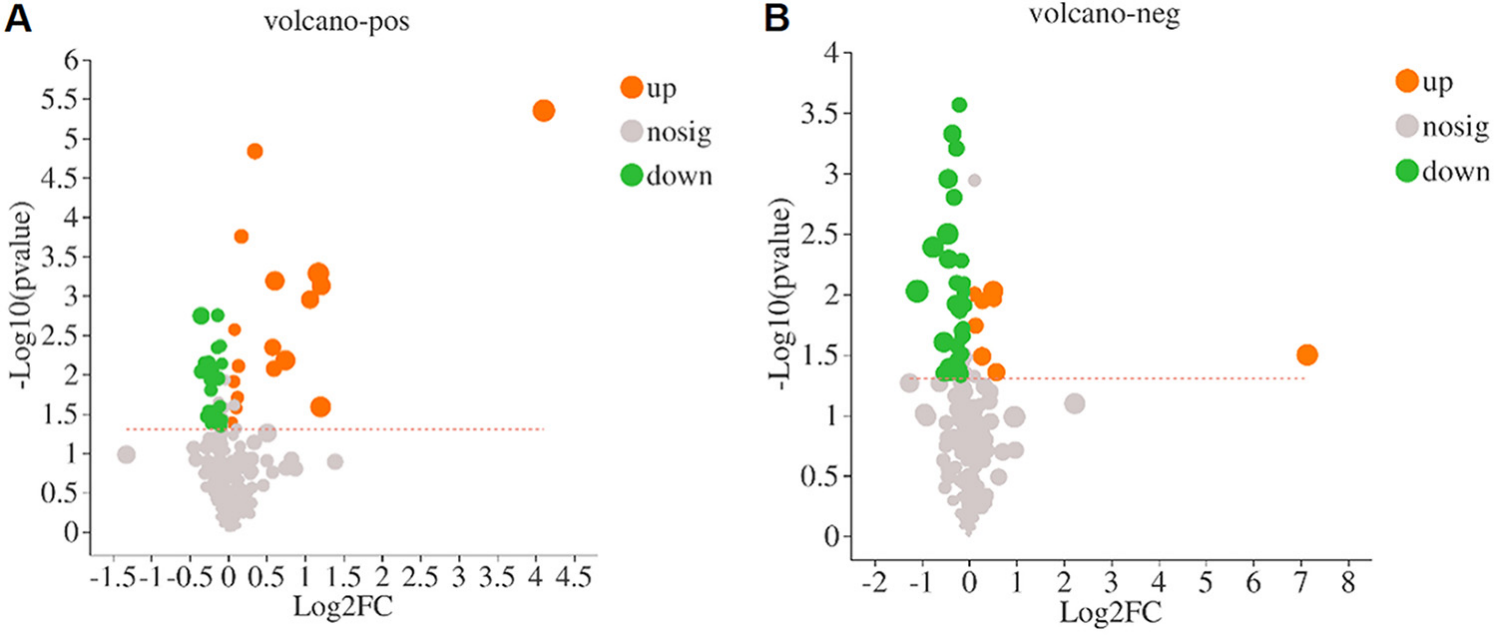
Volcanic maps of differentially accumulated metabolites in the WT and Δ*bolA* strains. Red dots denote upregulated metabolites, and green dots denote downregulated metabolites.

To compare the differentially accumulated metabolites between the WT and Δ*bolA* strains, a liquid chromatography-mass spectrometry (LC-MS) system was used for metabolite identification. A total of 82 differentially accumulated metabolites with known specific substances were screened. These differentially accumulated metabolites were defined as those with variable importance for projection (VIP) value >1 and *P* < 0.05 compared to the WT strain. Among the detected metabolites, 25 metabolites were upregulated, and 57 metabolites were downregulated. Among them, 17 metabolites were increased and 24 metabolites were decreased in positive mode, while 8 metabolites were increased and 33 metabolites were decreased in negative mode. The differentially accumulated metabolites were visualized by a volcano map (Fig. 4A). Based on the metabolomic analysis, five metabolites related to stress resistance and virulence were identified (agmatine, cadaverine, guanosine, flavin adenine dinucleotide [FAD], and d-biotin) (Table 2). We speculated that the downregulation of these five metabolites may be the factor leading to the weakening of the virulence and stress resistance of the Δ*bolA* strain of K. pneumoniae. However, further studies are still needed to explore the speculation.

**TABLE 2:** Differentially accumulated metabolites of virulence and stress resistance^*a*^

Name	Regulated type	FC (Δ*bolA*/WT)	VIP	*P* value
Agmatine	Down	0.87	1.28	0.0368
Cadaverine	Down	0.91	1.17	0.0045
Guanosine	Down	0.94	1.16	0.0454
FAD	Down	0.80	1.84	0.0016
d-Biotin	Down	0.79	1.53	0.0092

a Represented in Table 2 is the fold change of the Δ*bolA*/WT ratio for several metabolites related to virulence and stress resistance. FC, fold change. VIP, variable importance in projection. The VIP value was derived from the multivariate statistical analysis to characterize the contribution of the variable to the difference between the two groups.

### BolA protected K. pneumoniae cells against bile and oxidative stresses.

**FIG 5:**
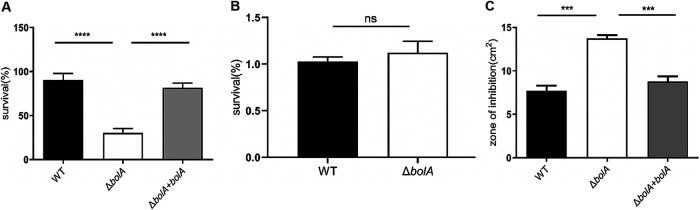
The role of BolA in the stress response (including bile, osmotic and oxidative stresses). (A) Bile resistance. The percentage of viable cells was calculated by plating the appropriate dilutions on LB agar plates containing bile (1.0%). (B) Resistance to osmotic stress. The ability of resistance to osmotic stress for the WT and Δ*bolA* strains was extrapolated by comparison to the numbers of viable cells on LB agar plates containing NaCl (0.5 M). (C) Resistance to hydrogen peroxide. The ability of strains to challenge hydrogen peroxide (30%) was measured by disc diffusion assay. ***, *P* < 0.001; ****, *P* < 0.0001; ns, not significant (*P* > 0.05) by *t* test.

To evaluate the role of K. pneumoniae BolA in intestinal colonization, bacteria underwent specific intestinal stresses associated with bile stress, osmotic stress, and oxidative stress. In the bile challenge experiment, we examined the ability of the strains to tolerate bile, and a significant difference was observed between the WT and Δ*bolA* strains (Fig. 5A). The WT, Δ*bolA*, and Δ*bolA+bolA* strains were grown in an LB medium containing 1% of oxbile, the ability of WT strain to grow in the 1% bile was 3-fold higher than that of Δ*bolA* strain, which was statistically significant (*P < *0.001). The Δ*bolA+bolA* strain restored the ability to tolerate bile stress. For the osmotic resistance assay, the results were surprising, and no significant difference was observed between the WT and the Δ*bolA* strains in an LB medium containing 0.5 M NaCl (Fig. 5B). An oxidative stress assay showed that the Δ*bolA* strain had a 1.79-fold greater sensitivity to 30% H_2_O_2_ (inhibition zone = 3.73 cm^2^) than the WT strain (inhibition zone = 7.69 cm^2^), which was statistically significant (*P < *0.001). The Δ*bolA+bolA* strain restored the ability to tolerate oxidative stress (Fig. 5C). Collectively, these results suggested that the BolA increased the survival rate of K. pneumoniae in bile and oxidative stresses.

### BolA promoted K. pneumoniae virulence in the G. mellonella larvae model of infection.

**FIG 6:**
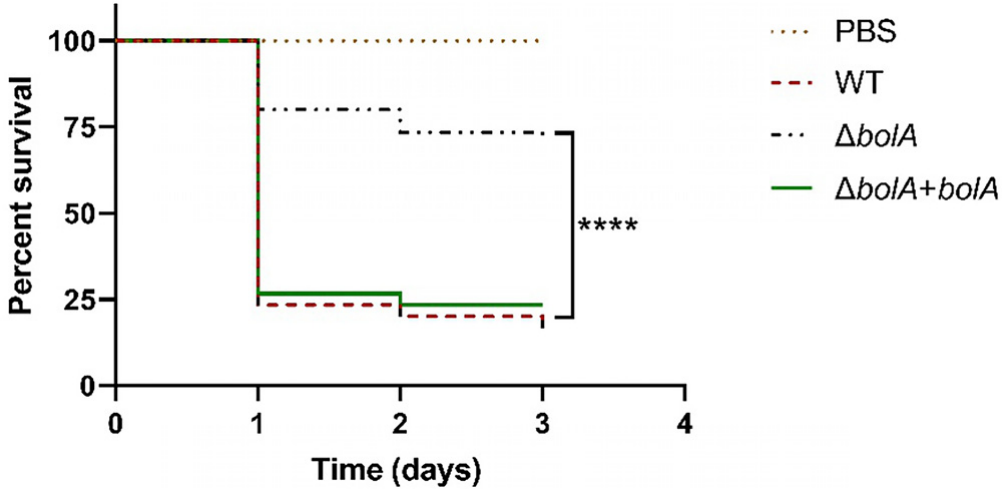
Survival of G. mellonella larvae following inoculation with K. pneumoniae strains. G. mellonella larvae were infected with PBS or 1 × 10^7^ CFU of WT, Δ*bolA*, or the Δ*bolA+bolA* strains. G. mellonella larvae infected with the Δ*bolA* strain had an enhanced survival rate compared to WT or the Δ*bolA+bolA* strains. Statistical significance was examined using Kaplan-Meier analysis with a log-rank (Mantel-Cox) test (****, *P* < 0.0001).

To evaluate the role of the BolA in the virulence of K. pneumoniae, we used the G. mellonella larvae for K. pneumoniae infection. G. mellonella larvae were injected with phosphate-buffered saline (PBS) or 2 × 10^5^ CFU/larva of the WT, Δ*bolA*, or Δ*bolA+bolA*, and the survival rate was daily monitored for 72 h (Fig. 6). The results of infection by the WT strain could be observed at 72 h of infection, with a decrease of about 83% of the initial larvae population. It was worth noting that the Δ*bolA* strain was found to increase by 53% the larval survival rate at 72 h after infection, compared with the survival rate of the WT strain. The Δ*bolA+bolA* strain was shown to restore the virulence to the level of the WT strain. These results indicated the essential role of *bolA* in the pathogenesis of K. pneumoniae in the G. mellonella larvae infection model.

### BolA promoted K. pneumoniae cell adhesion and *in vivo* colonization of mice.

**FIG 7:**
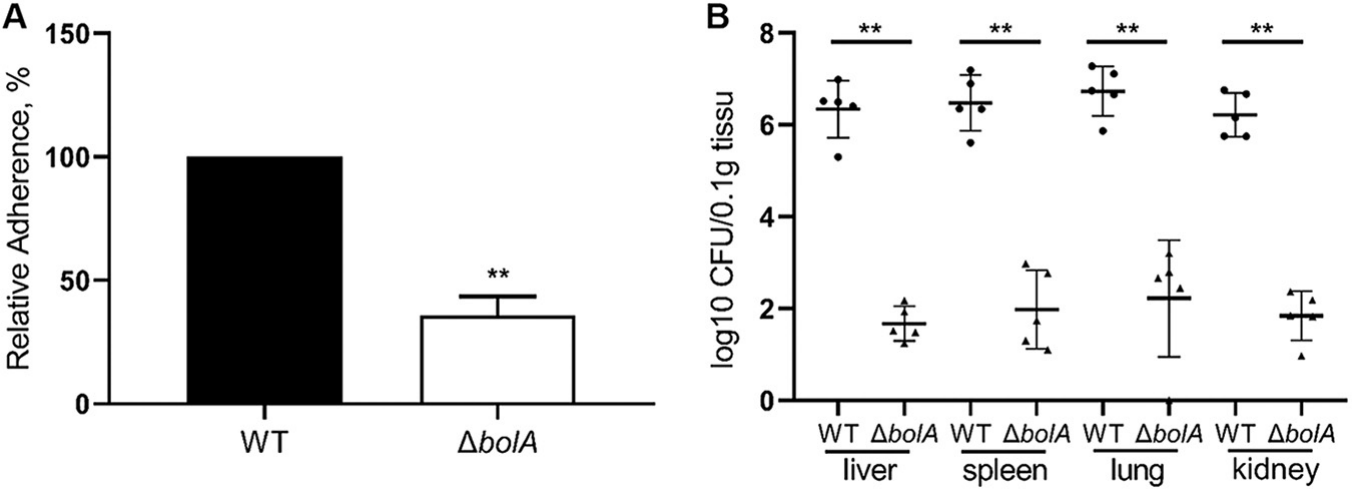
The role of BolA in the adherence to HCT116 cells and colonization ability in liver, lung, spleen, and kidney tissues of mice. (A) Adherence of the K. pneumoniae NTUH-K2044 and Δ*bolA* strains to HCT116 cells. The K. pneumoniae strains were grown to the logarithmic phase and added to HCT116 cells, incubated, and settled onto host cells for 1 h. Then, the bacteria were washed with PBS, released with Triton X-100, and plated onto LB agar for CFU counts. The adherence rate was calculated by dividing the number of adherent cells by the number of input cells. Briefly, 200 μL K. pneumoniae cells (OD595 = 0.5) were added to each well at a multiplicity of infection (MOI) of 200bacteria/cell. Considering that the MOI value was 200 rather than 1, then, the adherence rates of the *bolA* mutant strain were normalized to that of the WT strain (defined as 100%). **, *P* < 0.01 by *t* test. (B) 10-week-old female BALB/c mice were intraperitoneally injected with the WT and Δ*bolA* strains (5 mice for each group) at an inoculation dose of either 1 × 10^4^ CFU for 24 h. The bacterial burdens in the liver, spleen, lung, and kidney were determined for each mouse by quantitative plate count at 24 h after infection. Bacterial numbers (expressed as log 10 CFU) were standardized per 0.1 g of wet organ weight. Statistical significance was examined based on unpaired nonparametric Mann–Whitney U tests (**, *P* < 0.01).

The WT and Δ*bolA* strains were compared for their ability to adhere to HCT116 human colon cancer epithelial cells, and the adherence rate of the Δ*bolA* strain was normalized to that of the WT strain (100%) (Fig. 7A). The Δ*bolA* strain significantly decreased adherence compared with the WT strain, mounting 2.8-fold lesser adherence to HCT116 cells (*P < *0.01). Based on the results of stress challenge and cell adhesion experiments, we speculated that BolA may affect the adhesive capacity of K. pneumoniae in mice, and we performed *in vivo* verification. We used a mouse septicemia infection model to observe the colonization of bacteria in mouse tissues, including the liver, spleen, lung, and kidney. Ten-week-old female BALB/c mice were intraperitoneally injected with 2 × 10^4^ CFU in 500 μL of normal saline (NS). The mice were euthanized at 24 h postinoculation (hpi) and the liver, spleen, lung, and kidney bacterial burdens were determined. Given the *bolA* gene deletion, we anticipated that the colonization ability of the Δ*bolA* strain would be attenuated, and this was indeed observed. It was observed that the colonization ability of K. pneumoniae in the liver, spleen, lung, and kidney tissues of mice after the *bolA* deletion was significantly lower than the WT strain (*P < *0.01). In the liver, spleen, lung, and kidney at 24 hpi, the Δ*bolA* strain had colonization levels about 4 logs lower than K. pneumoniae NTUH-K2044 (Fig. 7B). These data indicated that the ability of the Δ*bolA* strain to survive and spread was attenuated in the mice.

**FIG 8:**
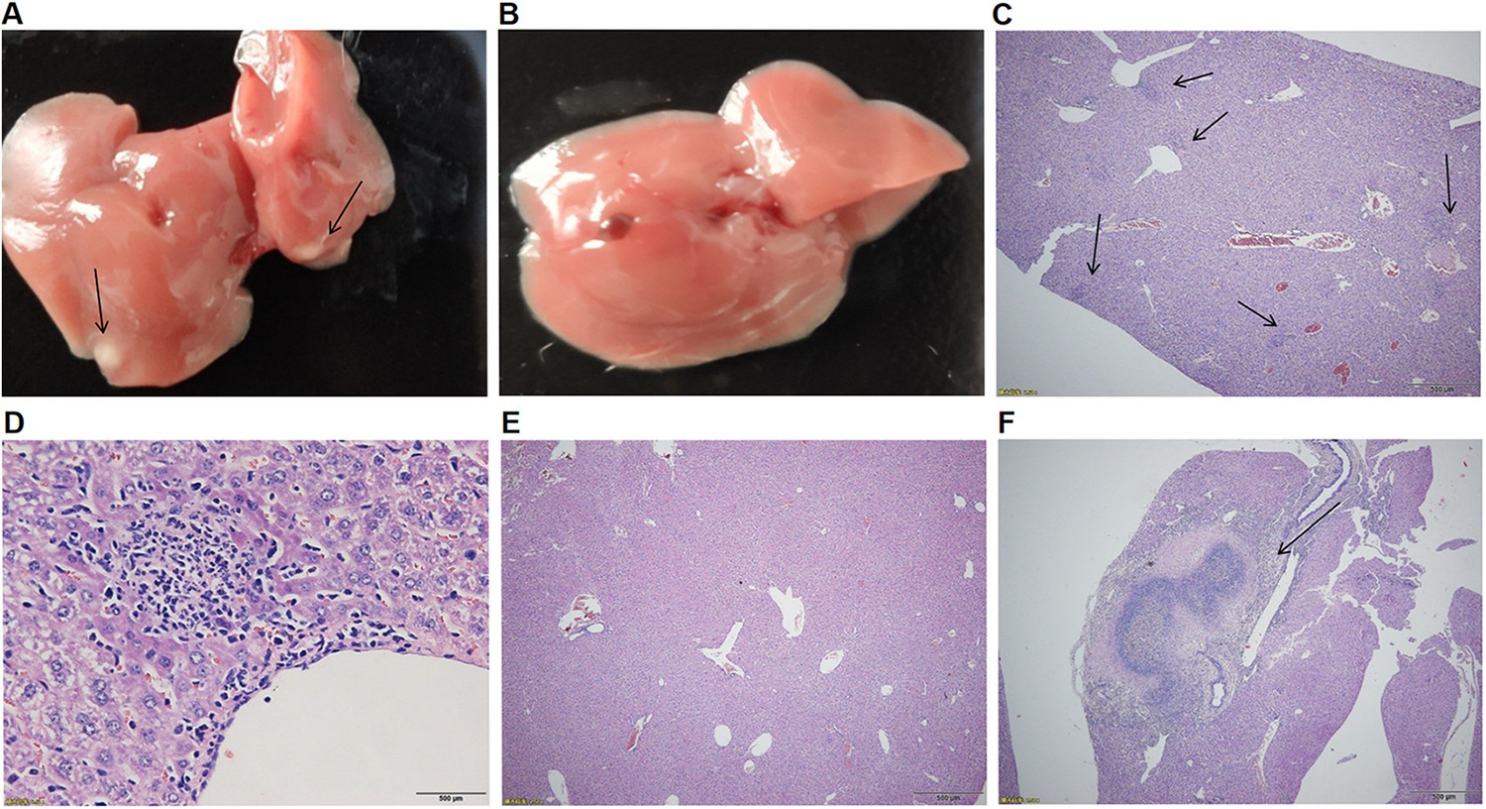
Histopathological examination of tissues from mice. Mice were inoculated with K. pneumoniae NTUH-K2044 or Δ*bolA* strains (2 × 10^3^ CFU). At 120 h after infection, liver tissues were taken for hematoxylin-eosin staining sections, and representative microscopic photos were selected for display (Fig. 8). In mice infected with the WT strain, macroabscesses (F, arrow) or microabscesses (C, arrow) were seen in the liver (magnification, ×40); The high-power field (magnification, ×400) shows the microabscesses composed of inflammatory cells (D). Furthermore, in mice infected with WT strain, the resected liver showed significant abscesses (A, arrow); No microabscess formation was observed in the liver (magnification, ×40) of mice infected with *bolA* mutant strain (E), and the resected liver shows no obvious abscess (B).

Histopathological evaluation of the livers from WT-infected mice at 120 h after infection revealed the frequent presence of either microabscesses (Fig. 8C, arrow) or macroabscesses (Fig. 8F, arrow). The high-power field (magnification, ×40) showed the microabscesses composed of inflammatory cells (Fig. 8D, arrow). In contrast, no microabscesses were observed in Δ*bolA*-infected mice (Fig. 8E). The numbers of liver abscesses were 14, 17, 12, and 3 (mean, 11.5) in 4 WT-infected mice, and 0, 0, 0, and 0 (mean, 0) in 4 Δ*bolA*-infected mice. Moreover, in WT-infected mice, gross liver abscesses were observed (Fig. 8A, arrow), and no liver abscesses were observed in Δ*bolA*-infected mice (Fig. 8B). In conclusion, BolA could promote the survival and growth of K. pneumoniae and then infect organs, causing liver abscesses in mice.

## DISCUSSION

In the current study, we characterized the K. pneumoniae NUTH-K2044 BolA protein and elucidated its role in several phenotypes associated with the process of cell morphology, siderophore production, biofilm formation, cell adhesion, tissue colonization, and liver abscess.

The amino acid sequences of E. coli K-12 W3110, *S.* Typhimurium SL1344, and K. pneumoniae NTUH-K2044 were used to search for the homologs of BolA in K. pneumoniae. It was observed that the protein sequence of BolA in K. pneumoniae and E. coli had a 91.4% similarity, that of *S.* Typhimurium SL1344 was 91.4% and the predicted three-dimensional (3D) structures of BolA proteins were nearly identical for the three species. These results indicated that BolA protein is highly conserved among E. coli, *S.* Typhimurium, and K. pneumoniae. We then investigated the effect of BolA on bacterial cell morphology and found that when K. pneumoniae NTUH-K2044 enters the stationary phase, the bacterial cells become rounder and shorter compared with the classical rod-shaped morphology. While the cell morphology of the Δ*bolA* strain cannot be maintained like the WT strain. We believed the rounder of bacterial cells results in a decrease in surface-to-volume ratio, and less surface area exposed to damaging or unfavorable environments. A similar result was also found in E. coli (10), and this result demonstrated that BolA is required for maintaining the cellular morphology of K. pneumoniae. Because BolA was identified as a stress-responsive protein and it is not clear whether BolA affects the growth rate of K. pneumoniae, we then measured the growth rate of the WT and Δ*bolA* strains in nutrient-rich LB and nutrient-restricted M9 medium. It could be found that there was no significant difference in growth rate between WT and Δ*bolA* strains in LB and M9 medium. Similar results were also found in *S.* Typhimurium (7). However, a previous study has shown that the deletion of *bolA* affects the growth rate of E. coli in a nutrient-rich medium (13). These results illustrated that BolA has disparate effects on the growth rate of different bacterial species and did not significantly impact the growth rate of K. pneumoniae.

Beyond that, studies have shown that bacterial biofilms are usually pathogenic, which often cause nosocomial infections, and that BolA is a transcription factor also involved in biofilm formation (20, 21). However, it is still unclear whether BolA affects the biofilms formation ability of K. pneumoniae. Therefore, we conducted biofilm formation experiments and found the biofilm formation ability of the Δ*bolA* strain was significantly lower than the WT strain, which was consistent with the result reported in the previous study (16). It is worth noting that BolA effectively promotes biofilm formation in K. pneumoniae NTUH-K2044 which might cause chronic infections and biofilm-related diseases. These results provided additional evidence that BolA plays distinct roles in different organisms.

Several studies have indicated the critical contribution of transcriptional regulators in bacterial adaptation and virulence (22–25). It is known that BolA is a bacterial transcription factor that has pleiotropic effects and promotes survival in different stresses (15). However, we still do not know the effects of BolA on the survival of K. pneumoniae under different stresses. We subsequently investigated the role of BolA in K. pneumoniae in response to bile and oxidative stresses, and BolA was found to increase the viability of K. pneumoniae under these two kinds of stresses. We also observed that the deletion of *bolA* in K. pneumoniae significantly reduces its adhesion rate in HCT116 cells, similar to what has been observed for the V. cholerae *bolA* homolog study (18). Moreover, we found that the ability to adhere to the intestinal epithelium in the *bolA* mutant strain was also attenuated. It is known that adhesion to intestinal epithelial cells could happen in the earlier stage of infection *in vitro* and appears to be important for K. pneumoniae pathogenesis. These data indicated that the absence of *bolA* reduced the ability of bacteria to protect themselves in the hosts. Thus, we believe that *bolA* is essential for the survival and infection of K. pneumoniae. These studies also pointed to a potential role of BolA in the virulence of K. pneumoniae and emphasized the significance of the identification of new transcriptional regulators to understand and overcome K. pneumoniae pathogenesis strategies. Besides, RNA-sequencing and RT-qPCR analysis suggested the direct effects of BolA are related to the induction of genes related to iron transport and the synthesis of siderophores. Through CAS agar plate experiments, we found that the Δ*bolA* strain exhibited a lesser halo than the WT strain in a CAS agar plate. These results indicated that BolA had a positive regulatory effect on siderophore production. To the best of our knowledge, this was the first time to determine the role of BolA in the capacity of iron uptake in K. pneumoniae, and we believe that BolA affected the production of siderophores by regulating the expression of the iron transport-related genes and siderophores synthesis gene *entE*.

To study the effects of BolA on K. pneumoniae proliferation and survival in the host, we chose the wax moth G. mellonella as the infected host. G. mellonella is an attractive infection model for human pathogens which has several advantages and has been increasingly used to study virulence mechanisms. In G. mellonella larvae killing experiments, our results suggested that the delete of the *bolA* gene significantly reduced the virulence of K. pneumoniae in the larvae of G. mellonella. The results showed that the K. pneumoniae *bolA* gene was absent, and the pathogenicity of the bacteria was decreased, similar to what has been observed for the *S.* Typhimurium BolA homolog study (7). We found that the Δ*bolA* strain significantly reduced the ability to colonize the liver, lung, spleen, and kidney organs of mice. Furthermore, histopathological examination showed that the formation of liver abscesses has not also been observed in the mice infected by the Δ*bolA* strain. The results revealed that the Δ*bolA* strain was defective in the formation of liver abscesses in mice. Based on the above results, we believed that BolA not only has effects on cell morphology, biofilm formation, iron uptake, the resistance to bile, and oxidation, but also affects the survival of K. pneumoniae in mice, and its ability to infect and cause disease is also reduced.

RNA-sequencing and metabolome assay were performed to evaluate the impact of BolA in global transcriptional regulation and compare the differentially accumulated metabolites among the WT and the Δ*bolA* strains, respectively. RNA-sequencing showed that the expression of T2SS, T6SS, siderophores synthesis, and iron transport-related virulence genes of the Δ*bolA* strain were significantly downregulated which was consistent with the results obtained in RT-qPCR. The metabolome analyses showed the metabolites related to virulence and stress resistance were downregulated in the Δ*bolA* strain (including biotin, spermine, cadaverine, guanosine, and FAD). It is worth noting that guanosine can become c-di-GMP and ppGpp when phosphorylated, which are the second messenger of bacteria, have a variety of physiological functions, among which the most important is to coordinate the stress response of bacteria (26, 27). Based on the above results, we hypothesized that virulence factor BolA may promote the virulence of K. pneumoniae by promoting the accumulation of five metabolites (biotin, spermine, cadaverine, guanosine, and FAD) and the expression of genes related to T2SS, T6SS, iron transport, siderophore, and ppGpp synthesis.

Taken together, we demonstrated for the first time that the K. pneumoniae BolA plays an important regulatory role in bacterial morphology, biofilm formation, and production of siderophore. When the *bolA* gene was deleted, reduced bile and oxidative stress resistance and reduced liver, spleen, lung, and kidney colonization were identified in mice. We proposed that BolA is a relevant player in K. pneumoniae virulence, contributing to the survival of this pathogen in a hostile environment during infection and providing new clues to the pathogenesis strategies of this organism. The current study also showed that the BolA is a good candidate as a therapeutic target against K. pneumoniae systemic infection. However, further studies are still needed to investigate other potential mechanisms associated with the effect of BolA on the virulence of K. pneumoniae and overcome K. pneumoniae strategies for successful infection and formation of liver abscess.

## MATERIALS AND METHODS

### Bacterial strains, plasmids, and growth conditions.

The bacterial strains and plasmids used are listed (see Table S1 in Supplemental File 1). All bacterial strains were stored at −80°C in an LB medium containing 25% glycerol. pKO3-Km was used to generate gene mutant strains through homologous recombination. Unless otherwise stated, K. pneumoniae and E. coli cultures were grown in LB medium, M9 (Na_2_HPO_4_ 6.8 g/liter; KH_2_PO_4_ 3.0 g/liter; NaCl 0.5 g/liter; NH_4_Cl 1.0 g/liter; and 0.6% glucose medium) medium or on LB agar at 30°C, 37°C or 43°C and supplemented with kanamycin (Km; 50 μg/mL or 25 μg/mL) where required. Bacterial growth was monitored by measuring the optical density at 595 nm (OD_595_).

For growth curves, overnight cultures were diluted 1:100 in LB or M9 medium and cultured at 37°C for 12 h. The OD_595_ values were measured by a microplate reader at hour intervals.

### Construction of the *bolA* gene deletion and complementation strains.

A *bolA* gene deletion strain (Δ*bolA*) of the K. pneumoniae NTUH-K2044 strain was constructed using the principle of homologous recombination as previously described (28). In brief, the left and right flanking DNA fragments of the *bolA* gene were amplified by PCR (using primers BolA-A/B and BolA-C/D) (see Table S2 in Supplemental File 1). Splicing overlap extension PCR (SOE-PCR) was used to amplify the DNA fusion fragments (using primers BolA-A/D). The fusion fragments and temperature-sensitive vector pKO3-Km were cut with NotI (New England Biolabs) and ligated with T4 DNA ligase (New England Biolabs). The recombinant plasmid pKO3-Km-Δ*bolA* was verified by PCR amplification and sequence determination (using primers pKO3-F/R). Then, the recombinant plasmid pKO3-Km-Δ*bolA* was introduced into NTUH-K2044 by electroporation (29, 30).

To construct the complementation strain (Δ*bolA+bolA*), the DNA fragment that contained the *bolA* coding sequence and promoter region (using primers BolA-HB-F/R) (see Table S2 in Supplemental File 1) was amplified by PCR. Then, the DNA fragment was cloned into the pGEM-T-easy-km. After that, the recombinant plasmid was transformed into the Δ*bolA* strain by electroporation (31). The gene deletion mutant and complementary strains were verified by PCR and sequence determination (using primers BolA-NF/NR).

### Scanning electron microscopy (SEM).

The SEM was performed to investigate the effect of BolA on bacterial cell morphology. Briefly, the supernatants were discarded after bacterial cultures were centrifuged. Bacterial cells were fixed overnight at 4°C in 2.5% glutaraldehyde. After washing them with PBS three times, they were dehydrated by gradient incubation and finally suspended in anhydrous ethanol. Drops of the bacterial suspension were applied to a glass coverslip, dried, and covered with chromium. Finally, bacterial cells were observed and photographed.

### CAS agar assays for iron uptake.

To elucidate the role of the BolA in the virulence of K. pneumoniae, CAS agar plates to detect the formation of siderophores were performed as described in the previously published articles (26, 32). In brief, it was prepared by first dissolving 60.5 mg of CAS powder (Yuanye Bio-Technology, China) in 50 mL of double-distilled water (ddH_2_O), and then adding 10 mL of 1 mM solution of FeCl_3_ (Aladdin Biochemical Technology Co., Ltd., Shanghai). Then, 72.9 mg hexadecyltrimethylammonium bromide (HDTMA; Yuanye Bio-Technology, China) was added to 40 mL ddH_2_O. Finally, 100 mL of the CAS stock solution made from the HDTMA solution was slowly poured into the CAS solution under stirring and autoclaved. Next, the freshly autoclaved 1.5% agar LB plate and CAS stock solution were mixed at a ratio of 9:1 to make a CAS agar plate. Then, 2 μL overnight bacterial cultures were inoculated on the CAS plate and cultivated at 28°C. The CAS plate was photographed after 48 h of cultivation. The assay was repeated three times.

### RNA sequencing.

To evaluate the impact of K. pneumoniae BolA in global transcriptional regulation. RNA sequencing was performed to compare the transcriptomic profiles of the WT and Δ*bolA* strains. The WT and Δ*bolA* strains were grown overnight in a 20 mL LB medium. The bacterial overnight cultures were diluted at 1:100 in fresh LB medium and grown to a stationary phase at 37°C. The bacteria were harvested by centrifugation and total RNAs were extracted using a Spin Column Bacteria Total RNA Purification kit (Sangon Biotech) according to the manufacturer’s instructions. The RNA quality and quantity were determined by 1.0% formaldehyde denaturing agarose gel electrophoresis and spectrophotometry in a NanoDrop ND-1000 machine, respectively. Illumina sequencing was performed at Novogene Bioinformatics Technology Co., Ltd. (Beijing, China), and the data were analyzed on the free online platform Novogene Cloud Platform. RNA-sequencing was carried out with three biological replicates for each strain.

### Metabolome assays.

To compare the differentially accumulated metabolites between the WT and Δ*bolA* strains, the metabolome assay was used for metabolite identification. The metabolome assay was performed at Majorbio Bio-pharm Technology Co., Ltd. (Shanghai, China). Briefly, the overnight cultures were centrifuged at 8000 × *g* for 10 min at 4°C. The supernatants were discarded. The cell pellets were then washed three times in 10 mL of PBS (4°C), resuspended in an extraction solvent consisting of methanol/water (4:1 vol/vol), and then a 6 mm grinding bead was added. After grinding for 6 min with a frozen tissue grinding instrument, the sample was extracted with low-temperature ultrasound for 30 min, and then the sample was placed at −20°C for 30 min The supernatant was centrifuged for 15 min and transferred to a tube for analysis. The sample extracts were analyzed by using an LC-MS system, and the data were analyzed on the free online platform of Majorbio Cloud Platform. The metabolome assays were performed with six biological replicates for each strain.

### RT-qPCR analysis.

RT-qPCR was performed to determine the expression levels of several differentially expressed genes (DEGs) identified in RNA-sequencing. The WT and Δ*bolA* strains were grown in LB medium to stationary phase, and then the bacterial cells were collected. The total RNA was extracted from harvested cells with a Spin Column Bacteria Total RNA Purification kit (Sangon Biotech, Shanghai). Then, the extracted RNA was reverse transcribed into cDNA using a TransScript All-in-One First-Strand cDNA Synthesis SuperMix (One-Step gDNA Removal). Finally, the RT-qPCR was performed using a Tip Green qPCR SuperMix (TransGen Biotech Co., Ltd.) in a Mastercycler ep realplex system (Eppendorf, Hamburg, Germany), with an initial incubation at 94°C for 30 s, followed by 40 cycles of 5 s at 94°C and 30 s at 60°C. The primers used for RT-qPCR are shown in Table S3 in Supplemental File 1. The internal control gene 16SrRNA was used to normalize the expression of each candidate gene. The threshold cycle (Ct) numbers were determined by the detection system software, and the data were analyzed based on the 2^−ΔΔCt^ method. The experiments were carried out in triplicate with three independent RNA samples.

### Biofilm formation assays.

The biofilm formation assays were performed to investigate whether BolA affects the biofilm formation ability of K. pneumoniae. The overnight bacterial cultures were diluted to approximately 10^8^ CFU/mL, and 200 μL of the diluted cultures were added to the 96-well polystyrene plate and incubated at 37°C for 48 h. Each well was washed with 200 μL PBS three times to remove planktonic bacteria. After staining with 0.1% crystal violet for 25 min, the crystal violet was washed thoroughly with PBS and the 96-well polystyrene plate was dried at room temperature. Then, 200 μL of anhydrous ethanol was added into each well to dissolve crystal violet and the biofilm thickness was estimated by measuring the absorbance of dissolved dye at 595 nm (OD_595_) (11). Each assay was performed in triplicate and repeated four times.

### Stresses challenge assays.

To evaluate the role of K. pneumoniae BolA in intestinal colonization, bacteria underwent specific intestinal stresses associated with bile stress, osmotic stress, and oxidative stress. Bile and osmotic stress assays were performed based on a previously described method (33). Briefly, bacterial cultures were grown separately until they reached an OD_595_ of 0.2 in LB medium. Then, the cultures were diluted 100000 times and 100 μL of the diluted cultures were plated onto LB agar plates containing NaCl (0.25 M, 0.5 M, or 0.75 M) and bile (1.0%, Sangon Biotech), respectively. The plates were incubated overnight at 37°C, and CFU was enumerated. The results were expressed as the ratio of the number of colonies obtained from the LB agar plate containing NaCl or bile to the number of colonies obtained from the LB agar plate. The experiments were performed at least three times.

For oxidative stress sensitivity assays (34), the exponential-phase bacterial cultures were diluted to an OD_595_ of 0.2 and were uniformly spread over an LB agar plate. Then, a sterile 6-mm paper disk (5 μL of 30% H_2_O_2_) was placed on the center of the agar surface. Next, the plates were incubated at 37°C for 24 h. The experiments were repeated at least three times.

### Adherence assays.

The WT and Δ*bolA* strains were compared for their ability to adhere to HCT116 human colon cancer epithelial cells. HCT116 human colon cancer epithelial cells were grown in RPMI 1640 medium supplemented with 10% fetal bovine serum (FBS), 100 U/mL penicillin, and 0.1 mg/mL streptomycin (35). Cell adhesion assays were performed mainly according to the methods described previously with minor modifications (36, 37). For adherence experiments, HCT116 cells were grown to confluent monolayers in 24-well plates, and then the cells were washed twice with Hank's balanced salt solution (HBSS). Followed by 200 μL of K. pneumoniae cells (OD_595_, 0.5) in FBS-free RPMI 1640 medium were added to each well and were settled onto host cells by centrifugation at 200 × *g* for 5 min. After incubating for 1 h at 37°C in 5% CO_2_ humidity, cells were washed 4 times with PBS, and then the bacteria were released by the addition of 0.5% Triton X-100. The recovered bacteria were quantified using serial dilution and plating onto LB agar. The adherence rate was calculated by dividing the number of adherent cells by the number of input cells. Briefly, 200 μL K. pneumoniae cells (OD595 = 0.5) were added to each well at a multiplicity of infection (MOI) of 200 bacteria/cell. Considering that the MOI value was 200 rather than 1, then, the adherence rates of the Δ*bolA* strain were normalized to that of the WT strain (defined as 100%). Assays were performed in duplicate and repeated at least three times.

### G. mellonella larvae killing assays.

To evaluate the role of the BolA in the virulence of K. pneumoniae, we used the G. mellonella larvae for K. pneumoniae infection. G. mellonella larvae killing assays were performed as described in the previously published articles (38, 39). Briefly, K. pneumoniae strains were prepared by harvesting exponential-phase bacterial cultures, washing twice in PBS, then adjusting to 1 × 10^7^ CFU. Groups of larvae (N = 10) were injected with 10 μL of working bacterial suspension at the last proleg using a hypodermic microsyringe. For each assay, a group of larvae was injected with PBS as a control. Injected larvae were placed in Petri dishes at 37°C for 72 h in the dark. The percent survival was recorded at 24 h intervals. G. mellonella larvae were considered dead if they did not respond to physical stimuli. Assays were repeated three times.

### Animal ethical statement.

This study was granted by the Institutional Animal Ethics Committee (IAEC). And all the assays were done maintaining the strict rules and regulations set. The approval number for the same was 20210008.

### Mice infection experiments.

The mouse septicemia infection model was used to observe the colonization of bacteria in mouse tissues to evaluate the role of the BolA in the virulence of K. pneumoniae. Ten-week-old female BALB/c mice (*n* = 5 in each group) were used for infections (40). Bacterial cells were collected by centrifugation and resuspended in sterile normal saline (NS). Mice were infected by intraperitoneal injection of the bacterial suspension (1 × 10^4^ CFU K. pneumoniae/mouse). After 24 h, the mice were euthanized and organs (including liver, spleen, kidney, and lung) were homogenized in NS, and then appropriate dilutions were plated onto LB agar for CFU counts.

Eight-week-old female BALB/c mice (4 per group) were infected by intraperitoneal injection with K. pneumoniae at a dose of 2 × 10^3^ CFU per mouse for 120 h (41). The livers were retrieved, fixed in 10% formalin, and embedded in paraffin blocks. The tissue sections were stained with hematoxylin-eosin and were imaged and quantified under a light microscope. The number of abscesses was quantified in five low-power fields (magnification, ×40).

### Statistical analyses.

All statistical analysis was performed using Prism (GraphPad). G. mellonella larvae survival was calculated using Kaplan-Meier analysis with a log-rank (Mantel-Cox) test. Differences in K. pneumoniae burden in tissues between groups were examined based on unpaired nonparametric Mann–Whitney U tests. Adherence assays, stress challenge assays, and iron uptake assays were analyzed by *t* test. Statistically significant was defined by *P* < 0.05 (*), *P* < 0.01 (**), *P* < 0.001 (***), and *P* < 0.0001 (****).

### Data availability.

The raw data of RNA-sequencing were submitted to the Short Read Archive (SRA) division of the GenBank repository under accession number PRJNA699464. The raw data of Metabolome have been deposited to the Metabolights database with accession number MTBLS2712.

## References

[1] Wen-Chien K. 2002. Community-acquired Klebsiella pneumoniae bacteremia: global differences in clinical patterns. Emerging Infectious Diseases 8:160–166. doi:10.3201/eid0802.010025. 11897067 PMC2732457

[2] Moradigaravand D, Martin V, Peacock SJ, Parkhil J. 2017. Evolution and epidemiology of multidrug-resistant Klebsiella pneumoniae in the United Kingdom and Ireland. mBio 8:e01976-16. doi:10.1128/mBio.01976-16. 28223459 PMC5358916

[3] Fu L, Huang M, Zhang X, Yang X, Liu Y, Zhang L, Zhang Z, Wang G, Zhou Y. 2018. Frequency of virulence factors in high biofilm formation bla(KPC-2) producing Klebsiella pneumoniae strains from hospitals. Microb Pathog 116:168–172. doi:10.1016/j.micpath.2018.01.030. 29360567

[4] Liu Y, Zhang H, Zhang X, Jiang N, Zhang Z, Zhang J, Zhu B, Wang G, Zhao K, Zhou Y. 2019. Characterization of an NDM-19-producing Klebsiella pneumoniae strain harboring 2 resistance plasmids from China. Diagn Microbiol Infect Dis 93:355–361. doi:10.1016/j.diagmicrobio.2018.11.007. 30552032

[5] Zhang B, Hu R, Liang Q, Liang S, Li Q, Bai J, Ding M, Zhang F, Zhou Y. 2022. Comparison of two distinct subpopulations of Klebsiella pneumoniae ST16 co-occurring in a single patient. Microbiol Spectr 10:e0262421. doi:10.1128/spectrum.02624-21. 35467408 PMC9241866

[6] Paczosa MK, Mecsas J. 2016. Klebsiella pneumoniae: going on the offense with a strong defense. Microbiol Mol Biol Rev 80:629–661. doi:10.1128/MMBR.00078-15. 27307579 PMC4981674

[7] Mil-Homens D, Barahona S, Moreira RN, Silva IJ, Pinto SN, Fialho AM, Arraiano CM. 2018. Stress response protein BolA influences fitness and promotes Salmonella enterica serovar Typhimurium virulence. Appl Environ Microbiol 84:e02850-17. doi:10.1128/AEM.02850-17. 29439986 PMC5881071

[8] Huynen MA, Spronk CA, Gabaldón T, Snel B. 2005. Combining data from genomes, Y2H and 3D structure indicates that BolA is a reductase interacting with a glutaredoxin. FEBS Lett 579:591–596. doi:10.1016/j.febslet.2004.11.111. 15670813

[9] Aldea M, Hernández-Chico C, de la Campa AG, Kushner SR, Vicente M. 1988. Identification, cloning, and expression of bolA, an ftsZ-dependent morphogene of Escherichia coli. J Bacteriol 170:5169–5176. doi:10.1128/jb.170.11.5169-5176.1988. 3053647 PMC211586

[10] Santos JM, Freire P, Vicente M, Arraiano CM. 1999. The stationary-phase morphogene bolA from Escherichia coli is induced by stress during early stages of growth. Mol Microbiol 32:789–798. doi:10.1046/j.1365-2958.1999.01397.x. 10361282

[11] Vieira HL, Freire P, Arraiano CM. 2004. Effect of Escherichia coli morphogene bolA on biofilms. Appl Environ Microbiol 70:5682–5684. doi:10.1128/AEM.70.9.5682-5684.2004. 15345459 PMC520865

[12] Adnan M, Morton G, Singh J, Hadi S. 2010. Contribution of rpoS and bolA genes in biofilm formation in Escherichia coli K-12 MG1655. Mol Cell Biochem 342:207–213. doi:10.1007/s11010-010-0485-7. 20480211

[13] Guinote IB, Moreira RN, Barahona S, Freire P, Vicente M, Arraiano CM. 2014. Breaking through the stress barrier: the role of BolA in Gram-negative survival. World J Microbiol Biotechnol 30:2559–2566. doi:10.1007/s11274-014-1702-4. 25038865

[14] Talib EA, Outten CE. 2021. Iron-sulfur cluster biogenesis, trafficking, and signaling: roles for CGFS glutaredoxins and BolA proteins. Biochim Biophys Acta Mol Cell Res 1868:118847. doi:10.1016/j.bbamcr.2020.118847. 32910989 PMC7837452

[15] Azam MW, Zuberi A, Khan AU. 2020. bolA gene involved in curli amyloids and fimbriae production in E. coli: exploring pathways to inhibit biofilm and amyloid formation. J Biol Res (Thessalon) 27:10. doi:10.1186/s40709-020-00120-7. 32566535 PMC7301969

[16] Dressaire C, Moreira RN, Barahona S, Alves de Matos AP, Arraiano CM. 2015. BolA is a transcriptional switch that turns off motility and turns on biofilm development. mBio 6:e02352-14–e02314. doi:10.1128/mBio.02352-14. 25691594 PMC4337573

[17] Moreira RN, Dressaire C, Barahona S, Galego L, Kaever V, Jenal U, Arraiano CM. 2017. BolA is required for the accurate regulation of c-di-GMP, a central player in biofilm formation. mBio 8:e00443-17. doi:10.1128/mBio.00443-17. 28928205 PMC5605933

[18] Fleurie A, Zoued A, Alvarez L, Hines KM, Cava F, Xu L, Davis BM, Waldor MK. 2019. A Vibrio cholerae BolA-like protein is required for proper cell shape and cell envelope integrity. mBio 10:e00790-19. doi:10.1128/mBio.00790-19. 31289173 PMC6747721

[19] Aldea M, Garrido T, Hernández-Chico C, Vicente M, Kushner SR. 1989. Induction of a growth-phase-dependent promoter triggers transcription of bolA, an Escherichia coli morphogene. EMBO J 8:3923–3931. doi:10.1002/j.1460-2075.1989.tb08573.x. 2684651 PMC402084

[20] Jamal M, Ahmad W, Andleeb S, Jalil F, Imran M, Nawaz MA, Hussain T, Ali M, Rafiq M, Kamil MA. 2018. Bacterial biofilm and associated infections. J Chin Med Assoc 81:7–11. doi:10.1016/j.jcma.2017.07.012. 29042186

[21] Vuotto C, Longo F, Pascolini C, Donelli G, Balice MP, Libori MF, Tiracchia V, Salvia A, Varaldo PE. 2017. Biofilm formation and antibiotic resistance in Klebsiella pneumoniae urinary strains. J Appl Microbiol 123:1003–1018. doi:10.1111/jam.13533. 28731269

[22] Storey D, McNally A, Åstrand M, Sa-Pessoa Graca Santos J, Rodriguez-Escudero I, Elmore B, Palacios L, Marshall H, Hobley L, Molina M, Cid VJ, Salminen TA, Bengoechea JA. 2020. Klebsiella pneumoniae type VI secretion system-mediated microbial competition is PhoPQ controlled and reactive oxygen species dependent. PLoS Pathog 16:e1007969. doi:10.1371/journal.ppat.1007969. 32191774 PMC7108748

[23] Dan P, Xuan L, Pin L, Xipeng Z, Mei L, Kewen S, Shuai C, Zhongshuang Z, Qiang H, Jingfu Q. 2018. Transcriptional regulation of galF by RcsAB affects capsular polysaccharide formation in Klebsiella pneumoniae NTUH-K2044. Microbiol Res 216:70–78. doi:10.1016/j.micres.2018.08.010. 30269858

[24] Disi L, Jinming F, Jingjie W, Long L, Li X, Feiyu L, Jing Y, Bei L. 2018. The fructose-specific phosphotransferase system of Klebsiella pneumoniae is regulated by global regulator CRP and linked to virulence and growth. Infect Immun 86:e00340-18. doi:10.1128/IAI.00340-18. 29844239 PMC6056848

[25] Wu CC, Wang CK, Chen YC, Lin TH, Jinn TR, Lin CT. 2014. IscR regulation of capsular polysaccharide biosynthesis and iron-acquisition systems in Klebsiella pneumoniae CG43. PLoS One 9:e107812. doi:10.1371/journal.pone.0107812. 25237815 PMC4169559

[26] Wang T, Cai Z, Shao X, Zhang W, Xie Y, Zhang Y, Hua C, Schuster SC, Yang L, Deng X. 2019. Pleiotropic effects of c-di-GMP content in Pseudomonas syringae. Appl Environ Microbiol 85:e00152-19. doi:10.1128/AEM.00152-19. 30850427 PMC6498148

[27] Hauryliuk V, Atkinson GC, Murakami KS, Tenson T, Gerdes K. 2015. Recent functional insights into the role of (p)ppGpp in bacterial physiology. Nat Rev Microbiol 13:298–309. doi:10.1038/nrmicro3448. 25853779 PMC4659695

[28] Hsieh P-F, Lin T-L, Lee C-Z, Tsai S-F, Wang J-T. 2008. Serum-induced iron-acquisition systems and TonB contribute to virulence in Klebsiella pneumoniae causing primary pyogenic liver abscess. J Infect Dis 197:1717–1727. doi:10.1086/588383. 18433330

[29] Chuang YP, Fang CT, Lai SY, Chang SC, Wang JT. 2006. Genetic determinants of capsular serotype K1 of Klebsiella pneumoniae causing primary pyogenic liver abscess. J Infect Dis 193:645–654. doi:10.1086/499968. 16453259

[30] Link AJ, Phillips D, Church GM. 1997. Methods for generating precise deletions and insertions in the genome of wild-type Escherichia coli: application to open reading frame characterization. J Bacteriol 179:6228–6237. doi:10.1128/jb.179.20.6228-6237.1997. 9335267 PMC179534

[31] Chen D, Zhao Y, Qiu Y, Xiao L, He H, Zheng D, Li X, Yu X, Xu N, Hu X, Chen F, Li H, Chen Y. 2019. CusS-CusR two-component system mediates tigecycline resistance in carbapenem-resistant Klebsiella pneumoniae. Front Microbiol 10:3159. doi:10.3389/fmicb.2019.03159. 32047485 PMC6997431

[32] Owen JG, Ackerley DF. 2011. Characterization of pyoverdine and achromobactin in Pseudomonas syringae pv. phaseolicola 1448a. BMC Microbiol 11:218. doi:10.1186/1471-2180-11-218. 21967163 PMC3207962

[33] Bharathi SV, Vasanth V, Amitabha M, Govindan R, Christiane F. 2012. Role of the two component signal transduction system CpxAR in conferring cefepime and chloramphenicol resistance in Klebsiella pneumoniae NTUH-K2044. PLoS One 7:e33777. doi:10.1371/journal.pone.0033777. 22496764 PMC3319533

[34] Hennequin C, Forestier C. 2009. oxyR, a LysR-type regulator involved in Klebsiella pneumoniae mucosal and abiotic colonization. Infect Immun 77:5449–5457. doi:10.1128/IAI.00837-09. 19786563 PMC2786449

[35] Guillaume LB, Jean-Félix S, Philippe G, Annick BD, Gobert AP, Annie G, Christine M, Hay AG, Francis B, Josée H. 2017. The NAG sensor NagC regulates LEE gene expression and contributes to gut colonization by Escherichia coli O157:H7. Front Cell Infect Microbiol 7:134. doi:10.3389/fcimb.2017.00134. 28484684 PMC5401889

[36] Hsu C-R, Pan Y-J, Liu J-Y, Chen C-T, Lin T-L, Wang J-T. 2015. Klebsiella pneumoniae translocates across the intestinal epithelium via Rho GTPase- and phosphatidylinositol 3-kinase/akt-dependent cell invasion. Infect Immun 83:769–779. doi:10.1128/IAI.02345-14. 25452552 PMC4294243

[37] Sahly H, Podschun R, Oelschlaeger TA, Greiwe M, Parolis H, Hasty D, Kekow J, Ullmann U, Ofek I, Sela S. 2000. Capsule impedes adhesion to and invasion of epithelial cells by Klebsiella pneumoniae. Infect Immun 68:6744–6749. doi:10.1128/IAI.68.12.6744-6749.2000. 11083790 PMC97775

[38] Kidd TJ, Mills G, Sá-Pessoa J, Dumigan A, Frank CG, Insua JL, Ingram R, Hobley L, Bengoechea JA. 2017. A Klebsiella pneumoniae antibiotic resistance mechanism that subdues host defences and promotes virulence. EMBO Mol Med 9:430–447. doi:10.15252/emmm.201607336. 28202493 PMC5376759

[39] Zhang Y, Wang X, Wang Q, Chen H, Li H, Wang S, Wang R, Wang H. 2021. Emergence of tigecycline nonsusceptible and IMP-4 carbapenemase-producing K2-ST65 hypervirulent Klebsiella pneumoniae in China. Microbiol Spectr 9:e0130521. doi:10.1128/Spectrum.01305-21. 34704778 PMC8549734

[40] Zong B, Zhang Y, Wang X, Liu M, Zhang T, Zhu Y, Zheng Y, Hu L, Li P, Chen H, Tan C. 2019. Characterization of multiple type-VI secretion system (T6SS) VgrG proteins in the pathogenicity and antibacterial activity of porcine extra-intestinal pathogenic Escherichia coli. Virulence 10:118–132. doi:10.1080/21505594.2019.1573491. 30676217 PMC6363058

[41] Hsu C-R, Chang I-W, Hsieh P-F, Lin T-L, Liu P-Y, Huang C-H, Li K-T, Wang J-T. 2019. A Novel role for the Klebsiella pneumoniae Sap (sensitivity to antimicrobial peptides) transporter in intestinal cell interactions, innate immune responses, liver abscess, and virulence. J Infect Dis 219:1294–1306. doi:10.1093/infdis/jiy615. 30476200 PMC6452313

