# Supplementary material for: Identification of the BolA Protein Reveals a Novel Virulence Factor in K. pneumoniae That Contributes to Survival in Host
Source: Microbiol Spectr. 2022 Sep 19;10(5):e00378-22. doi: 10.1128/spectrum.00378-22 (PMC9603091; doi:10.1128/spectrum.00378-22)
Supplement: Supplemental file 1 — Supplemental material. Download spectrum.00378-22-s0001.pdf, PDF file, 0.7 MB [file spectrum.00378-22-s0001.pdf]

Table S1. Bacterial strains and plasmids used in the current study

Bacterial strains [a]	Reference or source	Plasmids type [b]	Reference or source
E. coli DH5α	TransGen Biotech	pKO3-Km	(1)
K. pneumoniae NTUH-K2044	Laboratory stock	pGEM-T easy-km	(1)
△*bolA*	this study	pKO3-Km-△*bolA*	this study
△*bolA*+*bolA*	this study	pGEM-T easy-km-*bolA*	this study

[a] *E. coli* DH5α: cloning host; *K. pneumoniae* NTUH-K2044: hypervirulent *K. pneumoniae* with K1 serotype; △*bolA*: NTUH-K2044 with the deletion of *bolA* gene; △*bolA*+*bolA*: △*bolA* complementation with *bolA* gene.

[b] pKO3-Km: Km^r, suicide vector; pGEM-T easy-km: Km^r, cloning vector; pKO3-Km-△*bolA*: Km^r, suicide vector for the deletion of *bolA*; pGEM-T easy-km-*bolA*: Km^r, cloning vector containing *bolA*.

References

1. Xu L, Lin DS, Yang J, Li J, Li B. 2016. [Effect of Klebsiella pneumoniae KbvR regulator on bacterial biofilm formation and capsular synthesis]. Nan Fang Yi Ke Da Xue Xue Bao 36:1435-1439.

Table S2. Oligonucleotide primers used in this study

Primers	Sequence (5’-3’)
BolA-A	GTATGCGGCCGCGATGTTATAGCGCACGTTACCC
BolA-B	TTCTAAGCGATACTCCCTGCGAACGCTGCTCTAAGTTTTGCTT
BolA-C	AAGCAAAACTTAGAGCAGCGTTCGCAGGGAGTATCGCTTAGAA
BolA-D	GTATGCGGCCGCGTATTCGCCCGGAACGTAGTT
BolA-NF	GTTATCCGTCACAACGTCCCT
BolA-NR	ATACCGTATCCTGTAATGCTTCC
pKO3-F	GTTGAATATGGCTCATAACACCC
pKO3-R	TTGCTACGCCTGAATAAGTGA
BolA-HB-F	GTGTCGACAATCACAGTGCTCGGTCAACTC
BolA-HB-R	GAGTCCATGGTCTCAACGCATGGTGTTGTTAT

Table S3. The primers used for quantitative reverse-transcription PCR

Primers	Sequence (5'- 3')	Gene	Product size (bp)
pulH-F	TGGTGCTGCTGGCGTTTCC	*pulH*	101
pulH-R	GGGTGGATGATAATGCCGAAGA		
pulK-F	GTCTTCGTTCGGCTGCTGG	*pulK*	184
pulK-R	TAGACCTCGTCCTCCGCTCC		
pulE-F	CCGCACGGCATCATCCTT	*pulE*	102
pulE-R	CGACTTTGGCGTTGACCTGA		
vgrG-F	ATGAGTAGCGTGAAATCGTTG	*vgrG*	136
vgrG-R	CAATACGGTAGCGGAACG		
clpV-F	CCTGAAGTCCCGCTATGCC	*clpV*	125
clpV-R	CGAGTAAATCCACTGCCTTGTC		
afuA-F	GGTGGATTATGTCTCCTACGGC	*afuA*	122
afuA-R	CGGGATGCTGGCTGGTTT		
afuB-F	CTGCTGGCGGCGATGCTGAT	*afuB*	167
afuB-R	ATGCCCTGACCGACGGAACCCT		
fbpB-F	ATCTGTTTGTCGGCATCGC	*fbpB*	158
fbpB-R	ACAGGAATACCCACAGGAAGG		
fhuB-F	CGGCGTCATTCTGTTCGG	*fhuB*	124
fhuB-R	CAGCAGGATGGCGACTTTG		
FeCD-F	CGCAACTTTGAGCAGCGGATTA	*FeCD*	119
FeCD-R	ATGCGGCTCGCCAAGCGGGTTA		
relA-F	CGTGACTGGCTGAACCCT	*relA*	132

relA-R	CTGATGCCCAAATGCTCC		
spoT-F	TTCCAGGTGACCCGATTAT	*spoT*	125
spoT-R	CCCACTCAACCGCCATAA		
entE-F	TCGGTTCCTTCACCTACAC	*entE*	114
entE-R	TGCTTGCCCAGTCGTTCA		
16S rRNA-F	CGGTGAATACGTTCYCGG	*16S rRNA*	128
16S rRNA-R	GGWTACCTTGTTACGACTT		

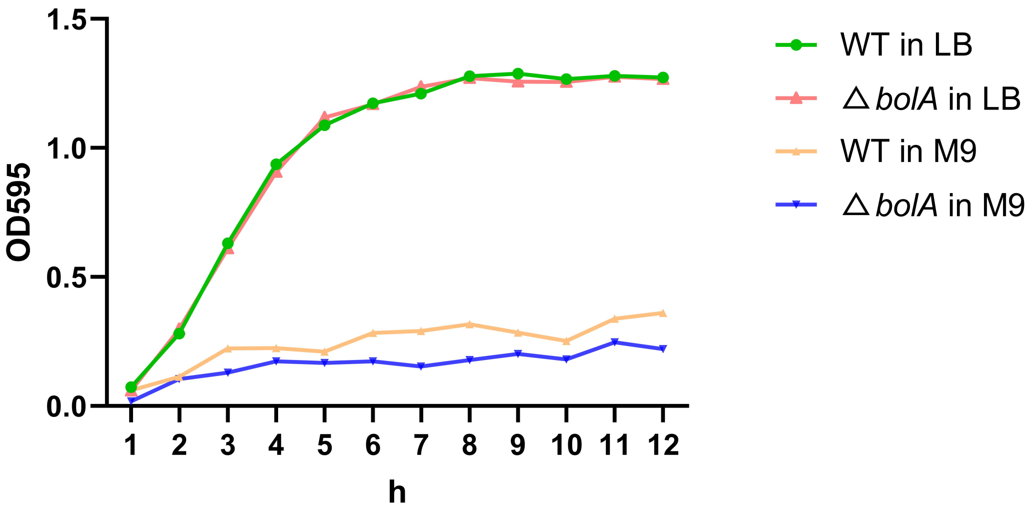

Fig S1. The growth kinetics of WT and △*bolA* strains in LB and M9 medium, and bacterial growth was monitored by measuring the optical density of the culture at 595 nm. Dots correspond to the mean values of three biological replicates for each strain. Each parent/mutant pair was compared by *t*-test with an assumption for unequal variance. During a 12-h period, It was found that the deletion of *bolA* gene did not affect the growth rate of *K. pneumoniae* in LB medium, and the growth rate of *bolA* mutant strain was reduced compared with the WT strain in the M9 medium. However, there was no significant difference in growth rate between these two strains ($p > 0.05$).
